# Alkaline Saliva

**Published:** 1900-03-15

**Authors:** M. H. Fletcher

**Affiliations:** Cincinnati, Ohio


### ALKALINE SALIVA.

BY M. H. FLETCHER, CINCINNATI, OHIO.

Read before the Ohio State Dental Society, December 6, 1899.

Miller says, "The saliva of cattle, sheep, dogs, rabbits, etc., is essentially strong in alkaline reaction." This may account for their perfect digestion, as well as their immunity from decayed teeth. This is not true of mankind, however, and in order to compensate for the physical defects of civilization, caused by eating prepared foods, the mouth should have conscious and continual attention. One demand seems to be that the saliva be artificially restored to its normal alkalinity.

Decay of the teeth may be arrested, or at least proceed very slowly, when the saliva is normally alkaline; the contrary is held to be true by some, but as stated, it is probably due to the strong alkaline saliva of animals that their teeth do not decay. The exception to this rule is found in domestic animals, or those kept as pets or in confinement and fed upon prepared foods.

Lactic acid is formed in the mouth by the action of bacteria upon all cooked starches, and is thought to be the great cause of decay of the teeth. A small portion of some of the sodium compounds dissolved in saliva quickly penetrates irregularities or cavities in the teeth, neutralizing the lactic acid of this ferment, as well as other acids found in the mouth.

The promptness with which sweets penetrate cavities in the teeth, producing pain and decay, gives a hint as how to try to arrest this process. The saliva is already in the places where the damage to the teeth is being done, and by utilizing it to dissolve a suitable alkali the seat of the trouble is reached at once.

Normal saliva is slightly alkaline, but the alkalinity is so weak that few mouths are capable of prompt recovery from an acid condition, neither is the alkalinity usually strong enough to counteract the acids of decay, hence it seems rational to endeavor to supply this deficiency.

The mucus, of which the saliva is partly composed, does not dissolve in water, ether, alcohol or dilute mineral acids, but dissolves readily in alkaline solutions; hence, any attempt to wash the saliva from the cavities in and about the teeth with anything but an alkaline solution is much like trying to remove grease without soap—the undisturbed globules of mucus remaining filled with micro-organisms.

The foregoing seems to indicate that an alkaline fluid is needed at the "portal" of the body for protection.

It is an accepted fact that the mouth is constantly inhabited by numerous germs, many of which are disease-producing. Within the space of an hour the particles of food remaining in the mouth become filled with microbes of many varieties. Saliva freighted with those germs being constantly swallowed may produce fermentation in the stomach, which results in eructations, sour stomach, headache, etc. Clinical experience shows that a small portion of a suitable alkali, allowed to dissolve in the saliva and swallowed, will quickly correct the disorder.

If the saliva is kept in its normally alkaline state, or in excess of normal, many of the acid-forming, fermentive, and putrefactive processes are prevented in the mouth; the saliva, being swallowed in this purer state, assists digestion instead of interfering with it.

The physiology of the mouth and stomach are so related that a remedy that will arrest putrefaction and fermentation in one organ may do so in the other, and mild alkalies are most accepted for this purpose. Some form of soda may be used, that of bicarbonate most frequently; this particular form is objectionable, however, because alkaline carbonates are utilized in producing lactic acid.

Considering the subject from the foregoing standpoint, the writer has for a period of years had it in mind to endeavor to meet these demands with a suitable mouth preparation, and for some months past has been experimenting to this end. The method of procedure was about as follows:

First, all things that are considered inefficient or objectionable in existing dentifrices and mouth washes were excluded. The pulverized chalk, sea-shells, pumice stone, cuttlefish bone, etc., seem always to have been used, and with this idea of polishing the teeth. But this is not what is needed, for their natural surface is the best. Anything that polishes also wears. The object, then, should be to keep them clean and no more. As far as the mechanical part of a dentifrice goes, if the earthy matters usually employed are coarse enough to really cleanse the teeth, saw-tooth and other abrasions may follow with their evil results, and if they are so fine as not to produce this effect little good from their use can follow. Dr. A. H. Peck's analysis of a number of mouth preparations proved that but one out of many had the essential feature, viz., that of alkalinity. The only soluble alkaline preparations known to the writer which have been much used for the mouth are those containing soap; but soap merely lubricates each particle of the powder and the bits of food about the teeth, causing the brush to slip easily over instead of removing them. Soap will relieve the mouth of oils and mucus, as it will the skin, but does not remove the tartar nor destroy bacteria. Toilet soaps, as a rule, are not strongly alkaline, therefore can not neutralize the acids of decay to any great degree, nor can they be relied upon to cleanse or purify sufficiently.

The foregoing facts necessitated a new start, the demands being as follows:

1. To find a mechanical cleanser which would not abrade, that a dentist might conscientiously recommend and a patient feel free to use without danger of injury.

2. The saliva must be utilized for dissolving an antiseptic and anti-acid, so that the cavities of decay would be penetrated by it, thus arresting caries and, if possible, prevent its recurrence by continued use. The acids found in the mouth, from any source, must also be counteracted.

3. Something must be found which will act upon the mucous membrane, for the purpose of keeping it in health or restoring it when it shows signs of disorder, and also to be a suitable treatment for lesions of the gums or mucous membrane.

4. With all these demands, it was necessary to find a combination the ingredients of which would be agreeable to each other and acceptable to the stomach, for the saliva passes in almost a constant stream to this organ, a fact too little considered in treatment of the mouth.

The results of months of experimenting without details are given. Mechanical cleansers and solvents were first investigated. Something softer than the softest ground ivory must be used. Wood fiber of many kinds was tried, resulting in making a very efficient dentifrice from cedar, boxwood, sandalwood and other very fine sawdusts; but each had its own peculiar flavor, many of which were undesirable—some giving up tannic acid, also objectionable, and the woody fiber remained about the teeth and under the tongue with a disagreeable effect.

Finally the raw, hard parts of various cereals were taken up with better results, and those of rice and Indian corn selected as being the best. These are to be used in the form of a coarse flour. This material is of itself a most efficient dentifrice, cleaning perfectly without injury.

The little blocks from the grains are like as much fine sand or emery in appearance, but are very much softer than the softest enamel or dentin, consequently can not abrade them; yet they are harder than the particles of food and of most deposits of tartar, and, being usable in much coarser condition than earthy powders, they are more efficient.

The argument has been advanced that this material, being starchy, would only assist the fermentive process in the mouth. This position is untenable from the fact that starch can not ferment until it is hydrated, and is only hydrated quickly by moist heat or caustic alkali.

As to the chemical part of the question, many things were considered and from various standpoints. Sodium borate and potassium chlorate were finally decided upon as being the best suited to all the conditions and to each other. They have proven clinically to be what they promised theoretically. The valuable therapeutic properties of both remedies are familiar to all physicians and dentists.

In the first place they are only slowly soluble in the saliva, and both act as the best of mechanical cleansers before they dissolve. They are both alteratives, tending to restore the mucous membrane when it is abnormal, and each acts complementary to the other.

The borate gives a decided alkaline reaction with litmus paper—first it is strong of the soda, changing into a sweetish, alkaline taste, which is very agreeable in effect. The meat packers of the United States alone use one thousand tons of borax yearly as an antiseptic. It is used in antiseptic surgery to quite an extent, being free from irritating properties, and is an excellent dressing for wounds, ulcers, abscesses, etc. Dr. Walb found that a 5 percent solution of it would keep fibrin perfectly fresh for nineteen days. Its value in medicine grows out of these properties, in addition to its peculiarly detergent, mildly-stimulating and alterative action upon the mucous membrane. In ulceration, diphtheria and other inflammations of the mouth crystals of it are allowed to dissolve slowly in the saliva. In solution it is also largely used in skin diseases. The internal dose is from fifteen to forty graims.

Potassium chlorate and its medical properties are well known; it gives a neutral reaction with litmus, and will promptly change acid saliva to neutral. Like borax, when locally applied, it is an alterative and stimulant to the mucous membrane, and is equally valuable for stomatitis and pharyngitis. When taken internally it is continually eliminated with the saliva, coming in perpetual contact with the mucous membrane, thus continuing its beneficial work until entirely excreted by the kidneys. The internal dose is from five to twenty grains. It is held by some to be an oxidizer, and in this way to purify and disinfect the mouth and stomach. Another reason is its saline taste, and its consequent stimulation to the flow of saliva; alkaline saliva seems an undoubted aid to digestion, and if it can be induced to flow and be kept alkaline, many stomach disorders will disappear.

The two remedies act most beautifully together, each as a complement to the other, without danger of overdosing. The mucous membrane, becoming inured and hardened to the action of these remedies, would naturally offer greater resistance to disease.

The materials being selected, they had now to be combined in the proper proportions to produce the desired results; clinical test was the standard selected to decide these points, and the preparation is now used very efficiently in the following proportions: Pulverized cereal, 75 percent; sodium borate, 17½ percent; potassium chlorate, 7½ percent. Orris and menthol to flavor, and saccharin to sweeten. The flavoring and sweetening can be varied to suit the taste.

It requires at least five grains at a time of any powder to be at all efficient in cleansing the mouth and teeth, and double the portion is better; in every five grains of the above formula there is one and one quarter grains of the combined remedies, this is sufficient to keep the saliva decidedly alkaline for some time after using. Its preservative and antiseptic properties are shown from the fact that ten grains of the powder taken into the mouth, and held until dissolved, then ejected into a test tube with a drachm of saliva, remained pure and unchanged for forty days, the test tube remaining unstopped.

It seems apparent that with this prescription teeth can be cleansed without injury, and that decay may be arrested; a small portion of a tablet made of it allowed to dissolve slowly in the mouth at frequent intervals would tend to arrest decay, if it did not prevent it.

There seems little question but that caries might be prevented if it were practicable to keep the teeth constantly bathed in a fresh, strong solution of the powder; if we assume this to be true, then the nearest approach that can be made to it the better for decaying teeth.

Dr. E. S. Talbot, of Chicago, who has been prescribing it for months, claims it to be a most valuable adjunct in the treatment of pyorrhea (interstitial gingivitis), and my own observations coincide with his.

There are those here who have had experience with this formula whose criticism would be acceptable, as will that of any one.

